# Two-Sample Tests Based on Data Depth

**DOI:** 10.3390/e25020238

**Published:** 2023-01-28

**Authors:** Xiaoping Shi, Yue Zhang, Yuejiao Fu

**Affiliations:** 1 Department of Computer Science, Mathematics, Physics and Statistics, University of British Columbia, Kelowna, BC V1V 1V7, Canada; 2 Department of Mathematics and Statistics, York University, Toronto, ON M3J 1P3, Canada

**Keywords:** hypothesis test, non-parametric tests, asymptotic distribution, multi-sample problem, data depth

## Abstract

In this paper, we focus on the homogeneity test that evaluates whether two multivariate samples come from the same distribution. This problem arises naturally in various applications, and there are many methods available in the literature. Based on data depth, several tests have been proposed for this problem but they may not be very powerful. In light of the recent development of data depth as an important measure in quality assurance, we propose two new test statistics for the multivariate two-sample homogeneity test. The proposed test statistics have the same $\chi^{2}\left(1\right)$ asymptotic null distribution. The generalization of the proposed tests into the multivariate multisample situation is discussed as well. Simulations studies demonstrate the superior performance of the proposed tests. The test procedure is illustrated through two real data examples.

## 1. Introduction

Multivariate two-sample homogeneity tests arise naturally in many applications. Consider two multivariate random samples $x_{1},x_{2},\ldots ,x_{m}$ and $y_{1},y_{2},\ldots ,y_{n}$ drawn from distributions *F* and *G*, respectively. The goal is to test $H_{0}$: F=G. The corresponding alternative hypothesis is that there are some discrepancies between the two distributions. The common formulation of the alternative hypothesis is based on location shift, scale change, or both. There is plenty of existing research on this topic. For example, the classical multivariate analysis of variance (MANOVA), an extension of the univariate analysis of variance (ANOVA), provides a general tool for testing multiple multivariate samples under normality and homogeneity of covariance matrices assumptions. The power of the MANOVA test may dramatically decrease when the assumptions are violated. Recently, some nonparametric methods were proposed such as the Cramér test, originally proposed by [1] and implemented in the *R* package *cramer*, and the Energy Distance test [2] implemented in the *R* package *energy*. Another work on nonparametric two-sample testing was given by [3], which draws connections between the multivariate Wasserstein test (implemented in the *R* package *otinference*) and the Energy Distance test.

In this paper, we consider another type of nonparametric test based on statistical depth. Let F(x) be a distribution in *d*-dimensional space and $x\in R^{d}$. Let D(x;F) denote a depth function which is a transformation from $R^{d}$ into [0,1]. A larger depth value indicates that the sample point is closer to the centrality of a given distribution or data cloud. There are some advantages of using statistical depth.

1. Depth is free of strong distributional assumptions. Unlike the MANOVA test, the depth-based test does not require the normality assumption. Moreover, there is a robust version of depth obtained with minimum covariance determinant (MCD) estimators [4,5]. The work in [6] listed four weak conditions for the depth function.
2. There are many different versions of depth that are easy to access. For example, the *R* package *ddalpha* includes Mahalanobis depth [7], spatial depth [8,9,10], projection depth [8,11], etc.
3. Depth can provide a ranking of multivariate data. The authors of [12,13] developed the so-called Kruskal–Wallis test based on the rank of univariate samples. Unlike univariate data, multivariate data lack a natural ranking. Recently, the authors of [14] generalized the Kruskal–Wallis test to a multivariate multisample homogeneity test and proposed a depth-based rank (DbR) test.

Statistical depth has become a popular and powerful tool in nonparametric inference and has been used in numerous fields. The work in [8] summarized four properties of depth: affine invariance, maximality at center, monotonicity relative to the deepest point, and vanishing at infinity. The author of [15] emphasized the advantage of depth for a location-scale model. The authors of [16] addressed the outlier detection problem based on depth. Further classification is treated in [17]. The work in [18] amended the halfspace depth and proposed a new illumination depth. For recent extensions to functional depth and regression depth, see [19,20,21].

In this paper, we mainly consider the improvement of the power of current statistical depth for a two-sample test. Ref. [7] approached the two-sample problem from the angle of quality assurance and proposed the quality index *Q*. The *Q* statistic is designed for the quality control problem, where *F* represents the “good” population, and *G* represents a future “check” population. In the context of quality control, *F* is naturally used as a reference distribution, and the *Q* statistic measures the overall “outlyingness” of *G* relative to the reference distribution *F*. We investigate the use of the quality index *Q* beyond the context of quality control but to the general two-sample homogeneity problem. In the general two-sample problem, either of the two samples can be regarded as a reference distribution; we need to consider pairwise quality indexes in order to capture the disparity between the two populations effectively.

The structure of the paper is outlined as follows. In Section 2, we propose two new depth-based test statistics constructed from pairwise quality indexes for the general two-sample homogeneity test. Interestingly, due to the asymptotical symmetry of the pairwise quality indexes, we show that the two proposed test statistics share the same asymptotic null distribution. In Section 3, simulation studies have demonstrated the superior performance of the proposed tests. In Section 4, we discuss the generalization in multivariate multisample cases. The test procedure is illustrated through two real data examples in Section 5. We draw conclusions and suggest future work in Section 6.

## 2. Main Results

The proposed tests are based on statistical depth. There are a variety of statistical depth functions proposed in the literature. In the present work, we focus on Mahalanobis depth, spatial depth, and projection depth, which are implemented in the *R* package *ddalpha*.

Consider any *d*-dimensional distribution *F* with mean μ and covariance matrix Σ. For any $x\in R^{d}$, the Mahalanobis depth of a sample point *x* is defined as

(1) $$MD\left(x;F\right)=\frac{1}{1+{\left(x-\mu \right)}^{\prime }\Sigma^{-1}\left(x-\mu \right)}.$$

If the underlying distribution *F* is unknown, it can be replaced by its corresponding empirical distribution, i.e., replacing μ and Σ with the sample mean and sample variance, respectively. The spatial depth is defined as

(2) $$SD\left(x;F\right)=\frac{1}{1+E(∥x-X∥)},$$

where *X* has a distribution *F*. The projection depth is defined as

(3) $$PD\left(x,F\right)=\frac{1}{1+O(x;F)},$$

where O(x;F), representing the outlyingness of a point *x*, is defined as

(4) $$O\left(x;F\right)=\underset{∥v∥=1}{\sup}\frac{|v^{⊤}x-Med\left(v^{⊤}X\right)|}{Med|v^{⊤}X-Med\left(v^{⊤}X\right)|},$$

where Med denotes the median function and *X* has a distribution *F*.

Our work was inspired by the *Q* statistic proposed by [7], so we review it in the following. For two *d*-dimensional distributions *F* and *G*, the *Q* statistic is defined as

(5) Q(F,G)=P{D(X;F)≤D(Y;F)|X∼F,Y∼G}.

When *F* and *G* are unknown, the *Q* statistic can be estimated by

(6) $$Q\left(F_{m},G_{n}\right)=\frac{1}{n}\sum_{i=1}^{n}R\left(y_{i};F_{m}\right),$$

where $F_{m}$ and $G_{n}$ represent the empirical distributions of *F* and *G*, respectively, $R(y_{i};F_{m})$ is defined as the sample proportion of $x_{1},x_{2},\ldots ,x_{m}$ having $D\left(x_{j};F_{m}\right)\leq D\left(y_{i};F_{m}\right)$. Here, we refer to $F_{m}$ as the reference distribution. Note that $Q\left(F_{m},G_{n}\right)\neq Q\left(G_{n},F_{m}\right)$ in general because a different reference distribution is used to compute the depth value. The authors of [7] showed that

(7) $${\left[\frac{1}{12}\left(\frac{1}{m}+\frac{1}{n}\right)\right]}^{-1}{\left[Q\left(F_{m},G_{n}\right)-\frac{1}{2}\right]}^{2}\overset{d}{\to }\chi^{2}\left(1\right).$$

Similarly, when we take $G_{n}$ as the reference distribution, we have

(8) $${\left[\frac{1}{12}\left(\frac{1}{m}+\frac{1}{n}\right)\right]}^{-1}{\left[Q\left(G_{n},F_{m}\right)-\frac{1}{2}\right]}^{2}\overset{d}{\to }\chi^{2}\left(1\right).$$

In practice, $Q(F_{m},G_{n})$ may perform better than $Q(G_{n},F_{m})$, and vice versa. We have the issue to choose a better one from the two pairwise quality indexes $Q(F_{m},G_{n})$ and $Q(G_{n},F_{m})$. Hence, we construct the following two new test statistics, the weighted average statistics $W_{m,n}\left(w_{1},w_{2}\right)$ and the maximum statistics $M_{m,n}$, to capture the disparity between the two populations efficiently:

(9) $$W_{m,n}\left(w_{1},w_{2}\right)={\left[\frac{1}{12}\left(\frac{1}{m}+\frac{1}{n}\right)\right]}^{-1}\frac{w_{1}{\left(Q\left(F_{m},G_{n}\right)-\frac{1}{2}\right)}^{2}+w_{2}{\left(Q\left(G_{n},F_{m}\right)-\frac{1}{2}\right)}^{2}}{w_{1}+w_{2}},$$

where $w_{1},w_{2}\geq 0$ and $w_{1}+w_{2}=1$, and

(10) $$M_{m,n}={\left[\frac{1}{12}\left(\frac{1}{m}+\frac{1}{n}\right)\right]}^{-1}\max\left\{{\left(Q\left(F_{m},G_{n}\right)-\frac{1}{2}\right)}^{2},{\left(Q\left(G_{n},F_{m}\right)-\frac{1}{2}\right)}^{2}\right\}.$$

If two distributions are different, at least one of ${\left(Q\left(F_{m},G_{n}\right)-\frac{1}{2}\right)}^{2}$ and ${\left(Q\left(G_{n},F_{m}\right)-\frac{1}{2}\right)}^{2}$ is large, so the weighted average statistics may be acceptable, and the maximum statistics are deemed to be superior. Note that prior information about the weights is needed to calculate the weighted average statistic $W_{m,n}\left(w_{1},w_{2}\right)$. When $w_{1}=1,w_{2}=0$ or $w_{1}=0,w_{2}=1$, we treat *G* or *F* as a reference distribution. One may set the weights according to the sample sizes. When m=n, one may use equal weights (i.e., $w_{1}=w_{2}=0.5$); otherwise, set $w_{1}=n/\left(m+n\right)=1-w_{2}$. In the general two-sample problem, both proposed test statistics can achieve better power than the original *Q* statistic under various alternative hypotheses, as demonstrated by our simulation studies. We recommend using the maximum statistic $M_{m,n}$ when no prior information on the weights is available.

For a general depth function, the authors of [6] studied sufficient conditions to guarantee the asymptotic null distributions in (7) and (8). Under the same conditions, we establish the asymptotically symmetrical properties of the pairwise quality indexes, which is a key result that leads to the asymptotic null distributions of the proposed test statistics. For the sake of the completeness of the presentation, we list these conditions below. Let $x_{1},\ldots ,x_{m}$ and $y_{1},\ldots ,y_{n}$ be independent samples from *F* and *G*, respectively, where *F* and *G* are any distribution functions. Let $F_{m}$ and $G_{n}$ be the corresponding empirical distribution function. For any point *x* and *y* and distribution *H* in $R^{d}$, let D(·;·) be a given (affine invariant) depth function with 0⩽D(x;H)⩽1.

(A1) $P\left\{y_{1}⩽D\left(Y;F\right)⩽y_{2}\right\}⩽C\left|y_{2}-y_{1}\right|$ for some *C* and any $y_{1},y_{2}\in \left[0,1\right]$.
(A2) $sup_{x\in R^{d}}\left|D\left(x;F_{m}\right)-D\left(x;F\right)\right|=o\left(1\right)$, almost surely, as m⟶∞.
(A3) $E(sup_{x\in R^{d}}|D\left(x;F_{m}\right)-D\left(x;F\right)\left|\right)=O\left(m^{-1/2}\right)$.
(A4) $E\left(\sum_{i}p_{iX}\left(F_{m}\right)p_{iY}\left(F_{m}\right)\right)=o\left(m^{-1/2}\right)$ if there exist $c_{i}$ such that $p_{iX}\left(F_{m}\right)>0$ and $p_{iY}\left(F_{m}\right)>0$ for $p_{iZ}\left(F_{m}\right):=P\left(D\left(Z;F_{m}\right)=c_{i}|F_{m}\right),i=1,2,\ldots ,m$.

Under conditions A1–A4, we show that $Q\left(F_{m},G_{n}\right)-1/2\approx 1/2-Q\left(G_{n},F_{m}\right)$. Based on the asymptotic symmetry property of the pairwise quality indexes, we show that both proposed test statistics are asymptotic pivotal and have a very simple $\chi^{2}\left(1\right)$ asymptotic null distribution, as stated in the following theorem. The proof of the theorem is given in Appendix A.

**Theorem** **1.** *Given two random samples $x_{1},x_{2},\ldots ,x_{m}$ and $y_{1},y_{2},\ldots ,y_{n}$ drawn from distributions F and G, respectively, let $F_{m}$ and $G_{n}$ be the corresponding empirical distribution function. Consider the two statistics $M_{m,n}$ and $W_{m,n}$ defined in *(9)* and *(10)*, respectively. If min(m,n)→∞ and m/n tends to a positive constant, under conditions A1–A4 and when null hypothesis is true, we have $W_{m,n}\left(w_{1},w_{2}\right)\overset{d}{\to }\chi^{2}\left(1\right)$ and $M_{m,n}\overset{d}{\to }\chi^{2}\left(1\right)$.*

## 3. Simulation Studies: Two-Sample Cases

In this section, simulation studies are conducted to examine the finite sample performance of the proposed tests in the general two-sample problem. We generate two random samples $x_{1},x_{2},\ldots ,x_{m}$ and $y_{1},y_{2},\ldots ,y_{n}$ from distributions *F* and *G*, respectively.

**Figure 1:**
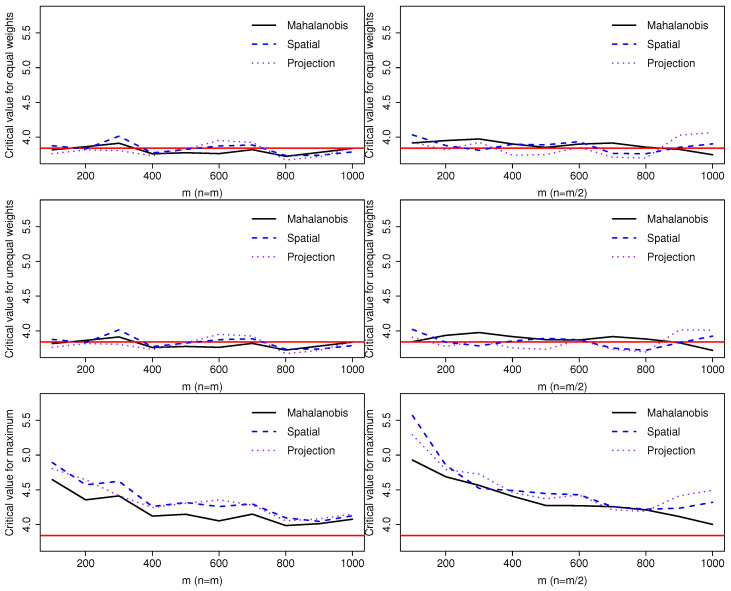
Comparison of empirical upper 95% quantiles of three statistics $W_{m,n}\left(0.5,0.5\right)$ (1st row), $W_{m,n}\left(\frac{n}{m+n},\frac{m}{m+n}\right)$ (2nd row), and $M_{m,n}$ (3rd row) for m=100,200,…,1000 and n=m (1st column) or n=m/2 (2nd column).

We first consider the Type I error of three statistics $W_{m,n}\left(\frac{1}{2},\frac{1}{2}\right)$, $W_{m,n}\left(\frac{n}{m+n},\frac{m}{m+n}\right)$, and $M_{m,n}$. Let $F=G=N(0,I_{2\times 2})$, where $N(0,I_{2\times 2})$ represents the bivariate normal distribution with mean vector 0 and two-by-two identity covariance matrix. We set m=100,200,…,1000 and n=m or m/2. By Theorem 1, all these statistics have the same $\chi^{2}\left(1\right)$ asymptotic null distribution. We consider the upper 95% quantile of $\chi^{2}\left(1\right)$, 3.84, and compare it with the empirical quantiles of three statistics for different configurations of *m* and *n* with 100,000 repetitions. Figure 1 shows the convergence rate of empirical quantiles to the theoretical one. When m=n, $W_{m,n}\left(0.5,0.5\right)$ and $W_{m,n}\left(\frac{n}{m+n},\frac{m}{m+n}\right)$ are the same and have a fast rate of convergence; otherwise, both rates are slower as shown in the first two rows. The convergence rate for $M_{m,n}$ is the slowest. Other simulations (not shown here for the sake of brevity) show that the quantile of $M_{m,n}$ approaches the nominal value when *m* exceeds 5000. Overall, all three depth functions lead to comparable results, except that $M_{m,n}$ has a faster rate of convergence when Mahalanobis depth is used for computation.

**Figure 2:**
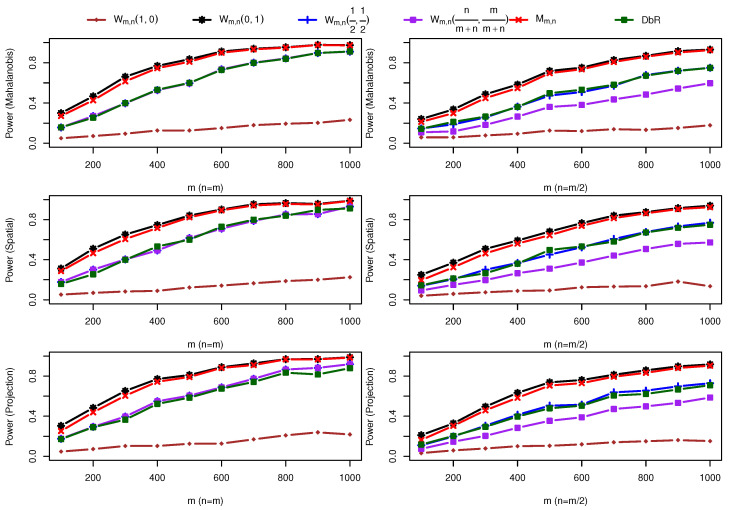
Power comparison under alternative hypothesis $F=N(0,I_{2\times 2})$ against $G=N(0,I_{2\times 2}+0.5{\tilde{I}}_{2\times 2})$ for m=100,200,…,1000. Different rows represent distinct depth functions applied, and two columns differentiate cases n=m and n=m/2.

**Figure 3:**
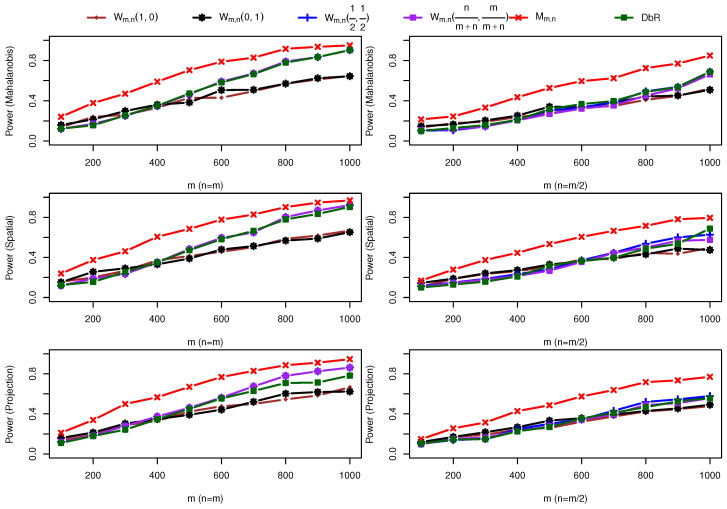
Power comparison under alternative hypothesis $F=N(0,I_{2\times 2})$ against $G=N({\left(0.35,0.35\right)}^{⊤},I_{2\times 2})$.

**Figure 4:**
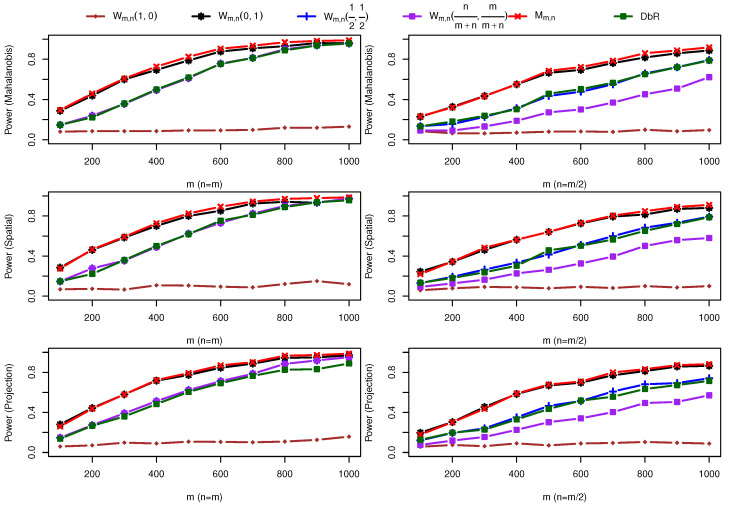
Power comparison under alternative hypothesis $F=N(0,I_{2\times 2})$ against $G=N({\left(0.3,0.3\right)}^{⊤},I_{2\times 2}+0.4{\tilde{I}}_{2\times 2})$.

Next, we consider the power of six test statistics: $W_{m,n}\left(1,0\right)$, $W_{m,n}\left(0,1\right)$, $W_{m,n}\left(0.5,0.5\right)$, $W_{m,n}\left(\frac{n}{m+n},\frac{m}{m+n}\right)$, $M_{m,n}$, and a depth-based rank (DbR) statistic [14]. The estimated power is calculated based on the simulated upper quantile α=0.05 for each statistic with Mahalanobis depth, spatial depth, and projection depth as the underlying depth function. The power study is conducted for the following three alternatives:

(1) Two bivariate normal distributions with a scale change: One sample is taken from $F=N(0,I_{2\times 2})$, and the other sample comes from $G=N(0,I_{2\times 2}+0.5{\tilde{I}}_{2\times 2})$, where ${\tilde{I}}_{2\times 2}=\left({\left(0,1\right)}^{⊤},{\left(1,0\right)}^{⊤}\right)$. Figure 2 shows the power comparison for different sample sizes and various depth functions with 1,000 repetitions. Compared with the difference between balanced sample size and unbalanced sample size, the difference among three depth functions is very small. The maximum statistic $M_{m,n}$ and one particular weighted statistic $W_{m,n}\left(1,0\right)$ have the largest power but $W_{m,n}\left(0,1\right)$ has the smallest power. In this case, *F* is preferred to be the reference distribution. In practice, since it is not clear which reference distribution should be used to capture the disparity between the two samples more effectively, the maximal statistic is recommended. It is interesting to see that the DbR statistic is comparable to the weighted statistic with equal weights.

(2) Two bivariate normal distributions with a mean change: One sample is taken from $F=N(0,I_{2\times 2})$ and the other sample comes from $G=N({\left(0.35,0.35\right)}^{⊤},I_{2\times 2})$. Different from the first scenario, Figure 3 shows that the maximum statistic $M_{m,n}$ outperforms all other five statistics to detect a location shift between the two samples.

(3) Two bivariate normal distributions with both mean and scale changes: One sample is taken from $F=N(0,I_{2\times 2})$, and the other sample is drawn from $G=N({\left(0.3,0.3\right)}^{⊤},I_{2\times 2}+0.4{\tilde{I}}_{2\times 2})$. Figure 4 shows that $M_{m,n}$ and $W_{m,n}\left(0,1\right)$ are comparable, and both outperform all other four test statistics.

**Figure 5:**
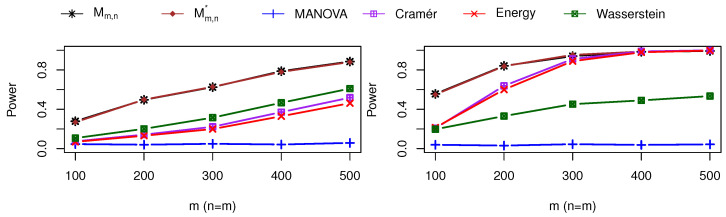
Power comparison under alternative hypothesis: two-component multivariate normal distributions (**left** panel) and multivariate t distributions (**right** panel).

We also compare the Mahalanobis-based maximum test denoted as $M_{m,n}$ and its robust version obtained with MCD estimators denoted as $M_{m,n}^{*}$, MANOVA test, Cramér test, Energy test, and Wasserstein test for different m=n=100,200,…,500 under two alternative hypotheses. For the first alternative hypothesis shown in the left panel of Figure 5, in the first sample, we draw 50% data from $N(0,I_{2\times 2})$ and 50% data from $N({\left(1,1\right)}^{⊤},I_{2\times 2})$, and in the second sample, we draw 95% data from $N(0.5+0,I_{2\times 2})$ and 5% data from $N(1+0,I_{2\times 2})$. Mahalanobis-based tests outperform all other four test statistics. The MANOVA test has almost no power.

For the second alternative hypothesis shown in the right panel of Figure 5, in the first sample, we draw a multivariate t distribution with mean 0, scale matrix $I_{2\times 2}$, and degrees of freedom of 2. In the second sample, we used a multivariate t distribution with mean 0, scale matrix $I_{2\times 2}+0.6{\tilde{I}}_{2\times 2}$, and degrees of freedom of 3. The Mahalanobis-based tests still perform the best, while the Cramér test and the Energy test also perform the same for large sample sizes. The MANOVA has no power for non-normal data.

## 4. Multisample Comparison

As demonstrated in the last section, the maximal statistic $M_{m,n}$ is more powerful than the weighted statistic in a two-sample comparison, especially against location shift alternatives. In this section, we extend the maximum statistic $M_{m,n}$ for comparing multisample multivariate distributions. Consider *k* random samples that are drawn from distributions $F^{\left(j\right)}$ with sample sizes $n_{j}$ and empirical distribution $F_{n_{j}}^{\left(j\right)}$, for j=1,2,…,k. The generalized maximum statistic for *k*-sample comparison is

(11) $$M_{n_{1},\ldots ,n_{k}}=\max_{1\leq i,j\leq k,i\neq j}{\left[\frac{1}{12}\left(\frac{1}{n_{i}}+\frac{1}{n_{j}}\right)\right]}^{-1}{\left[Q\left(F_{n_{i}}^{\left(i\right)},F_{n_{j}}^{\left(j\right)}\right)-\frac{1}{2}\right]}^{2}.$$

Similar to the proof of Theorem 1, for x>0 we have

(12) $$P\left(M_{n_{1},\ldots ,n_{k}}\leq x\right)\to P\left\{\max_{1\leq i<j\leq k}{\left(c_{i,j}Z_{i}+{\tilde{c}}_{i,j}Z_{j}\right)}^{2}\leq x\right\},$$

where $Z_{1},Z_{2},\ldots ,Z_{k}$ are independent from N(0,1), $c_{i,j}=\lim n_{i}^{-1/2}{\left(n_{i}^{-1}+n_{j}^{-1}\right)}^{-1/2}$, and ${\tilde{c}}_{i,j}=\lim n_{j}^{-1/2}{\left(n_{i}^{-1}+n_{j}^{-1}\right)}^{-1/2}$ with $c_{i,j}^{2}+{\tilde{c}}_{i,j}^{2}=1$.

When k=3, we have

$$\begin{array}{c} M_{n_{1},n_{2},n_{3}}=\max\{{\left[\frac{1}{12}\left(\frac{1}{n_{1}}+\frac{1}{n_{2}}\right)\right]}^{-1}{\left[Q\left(F_{n_{1}}^{\left(1\right)},F_{n_{2}}^{\left(2\right)}\right)-\frac{1}{2}\right]}^{2},[{\left(\frac{1}{12}\left(\frac{1}{n_{1}}+\frac{1}{n_{3}}\right)\right]}^{-1}{\left[Q\left(F_{n_{1}}^{\left(1\right)},F_{n_{3}}^{\left(3\right)}\right)-\frac{1}{2}\right]}^{2},\\ \frac{1}{12}\left(\frac{1}{n_{2}}+\frac{1}{n_{1}}\right){]}^{-1}{\left[Q\left(F_{n_{2}}^{\left(2\right)},F_{n_{1}}^{\left(1\right)}\right)-\frac{1}{2}\right]}^{2},[{\left(\frac{1}{12}\left(\frac{1}{n_{2}}+\frac{1}{n_{3}}\right)\right]}^{-1}{\left[Q\left(F_{n_{2}}^{\left(2\right)},F_{n_{3}}^{\left(3\right)}\right)-\frac{1}{2}\right]}^{2},\\ \frac{1}{12}\left(\frac{1}{n_{3}}+\frac{1}{n_{2}}\right){]}^{-1}{\left[Q\left(F_{n_{3}}^{\left(3\right)},F_{n_{2}}^{\left(2\right)}\right)-\frac{1}{2}\right]}^{2},\left[{\left(\frac{1}{12}\left(\frac{1}{n_{3}}+\frac{1}{n_{1}}\right)\right]}^{-1}{\left[Q\left(F_{n_{3}}^{\left(3\right)},F_{n_{1}}^{\left(1\right)}\right)-\frac{1}{2}\right]}^{2}\right\}. \end{array}$$

and

(13) $$P\left(M_{n_{1},n_{2},n_{3}}\leq x\right)\to P\left\{{\left(c_{1,2}Z_{1}+{\tilde{c}}_{1,2}Z_{2}\right)}^{2}\leq x,{\left(c_{1,3}Z_{1}+{\tilde{c}}_{1,3}Z_{3}\right)}^{2}\leq x,{\left(c_{2,3}Z_{2}+{\tilde{c}}_{2,3}Z_{3}\right)}^{2}\leq x\right\}.$$

**Figure 6:**
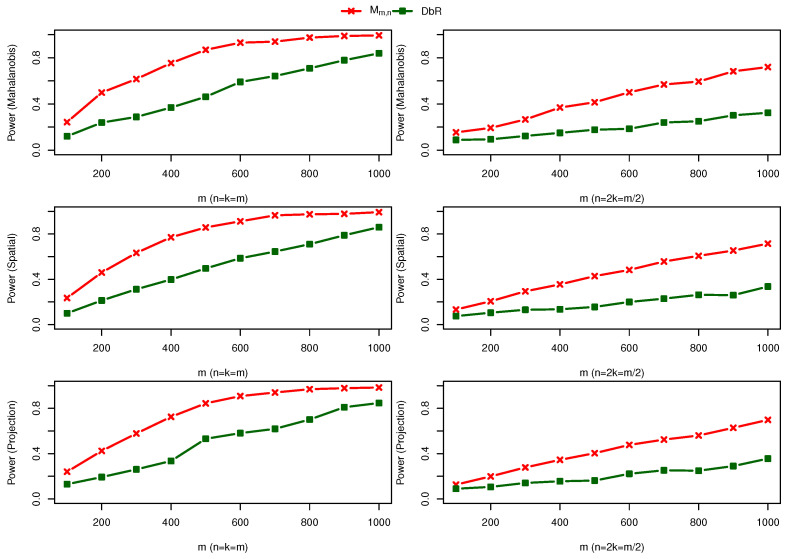
Power comparison of two statistics $M_{m,n}$ and DbR in [14] under alternative hypothesis $F^{\left(1\right)}=F^{\left(2\right)}=N\left(0,I_{2\times 2}\right)$ and $F^{\left(3\right)}=N\left({\left(0,0\right)}^{⊤},I_{2\times 2}+0.5{\tilde{I}}_{2\times 2}\right)$.

**Figure 7:**
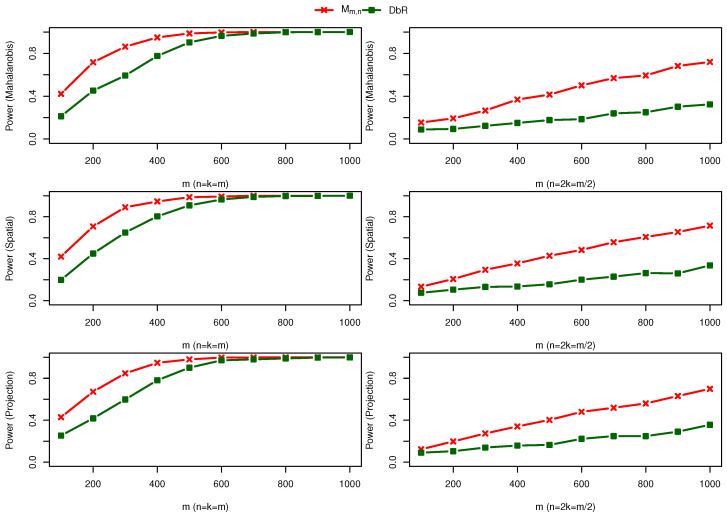
Power comparison of two statistics $M_{m,n}$ and DbR in [14] under alternative hypothesis $F^{\left(1\right)}=N\left(0,I_{2\times 2}\right)$, $F^{\left(2\right)}=N\left({\left(0.3,0.3\right)}^{⊤},I_{2\times 2}\right)$, and $F^{\left(3\right)}=N\left({\left(0,0\right)}^{⊤},I_{2\times 2}+0.5{\tilde{I}}_{2\times 2}\right)$.

The asymptotic properties of the generalized maximum statistic for *k*-sample comparison are left for future research. Here, we conduct simulation studies to evaluate the power performance of the maximum statistic $M_{m,n,k}$, and the DbR statistic. The estimated power is based on the simulated upper quantile α=0.05 for each statistic with Mahalanobis depth, spatial depth, or projection depth as the underlying depth function. We consider the following two alternatives:

(1) Three bivariate normal distributions: Two samples are taken from $F^{\left(1\right)}=F^{\left(2\right)}=N\left(0,I_{2\times 2}\right)$, and the third sample comes from $F^{\left(3\right)}=N\left({\left(0,0\right)}^{⊤},I_{2\times 2}+0.5{\tilde{I}}_{2\times 2}\right)$. Figure 6 compare the power of two statistics $M_{m,n,k}$ and DbR statistic for m=100,200,…,1000, three depth functions and two combinations n=k=m and n=2k=m/2 for 1000 repetitions. We can see that the maximal statistics $M_{m,n,k}$ outperforms the DbR statistic.

(2) Three distinguished bivariate normal distributions: $F^{\left(1\right)}=N\left(0,I_{2\times 2}\right)$, $F^{\left(2\right)}=N\left({\left(0.3,0.3\right)}^{⊤},I_{2\times 2}\right)$, and $F^{\left(3\right)}=N\left({\left(0,0\right)}^{⊤},I_{2\times 2}+0.5{\tilde{I}}_{2\times 2}\right)$. Figure 7 shows that the maximal statistic $M_{m,n,k}$ outperforms the DbR statistic for all the cases considered.

In summary, our simulation studies show that the maximal statistic $M_{m,n,k}$ is more powerful than the DbR statistic under various alternatives in three-sample comparisons. The choice of depth function does not significantly change the performance of the tests.

## 5. Analysis of Two Real-Data Sets

In this section, we illustrate our proposed maximum statistic for three-sample comparison in the analysis of two real-data examples.

### 5.1. Beans Data

In industry, automatic methods are required for testing. The authors of [22] analyzed seven different kinds of beans: Barbunya, Bombay, Cali, Dermason, Horoz, Seker and Sira, where 12-dimensional and 4-shape features were obtained for comparison. It is interesting to see whether our proposed test can distinguish Dermason and Sira because the difference between them only lies in the end: round and flat. There are 3546 Dermason beans and 2636 Sira beans. Those two features, perimeter and major axis length, may describe the difference well and are included here for a two-sample comparison.

To visualize the dispersion of distributions of two different kinds of beans, we compare scale curves proposed by [23] and implemented in the *R* package *DepthProc*, which allows us to compare the scale of different distributions graphically. Let $D_{\alpha }\left(F\right)$ be the α-trimmed region with respect to distribution *F*, that is,

$$D_{\alpha }\left(F\right)=\left\{x\in R^{d}:D\left(x;F\right)\geq \alpha \right\}.$$

**Figure 8:**
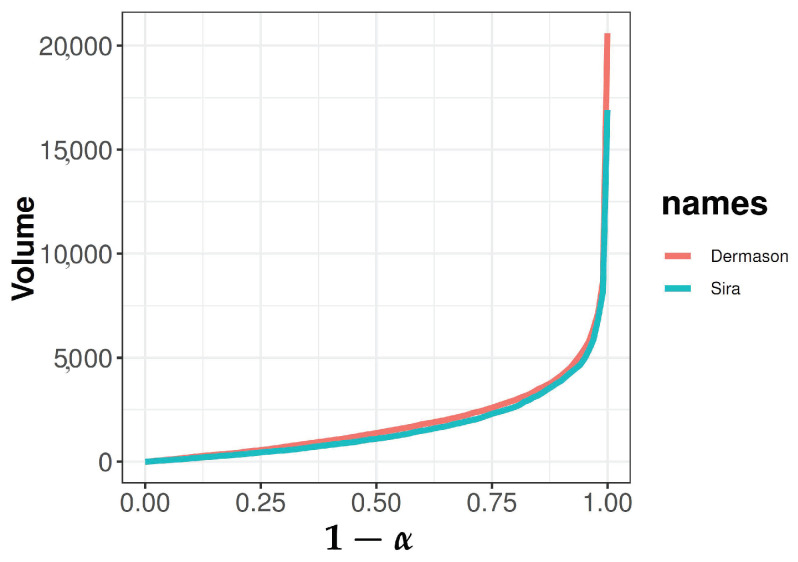
Scale curves for two kinds of beans under Mahalanobis depth.

Similarly, we have sample versions of $D(x;F_{m})$ and $D_{\alpha }\left(F_{m}\right)$. Let $V(\alpha ;F_{m})$ be the volume of convex region $D_{\alpha }\left(F_{m}\right)$. Define a sample scale curve by taking the plot of 1−α versus the volume $V(\alpha ;F_{m})$. The faster-growing scale curve is associated with a larger scale of distribution. Figure 8 presents the scale curves for the three species of iris under Mahalanobis depth.

We can see that the scale curves of the two kinds of beans are slightly different, which requires confirmation through a formal statistical hypothesis test. In fact, the asymptotic *p*-value by (13) is also zero for each depth function. This is also true for MANOVA, Cramér, Energy, and Wasserstein tests.

### 5.2. Egyptian Skulls Data

**Figure 9:**
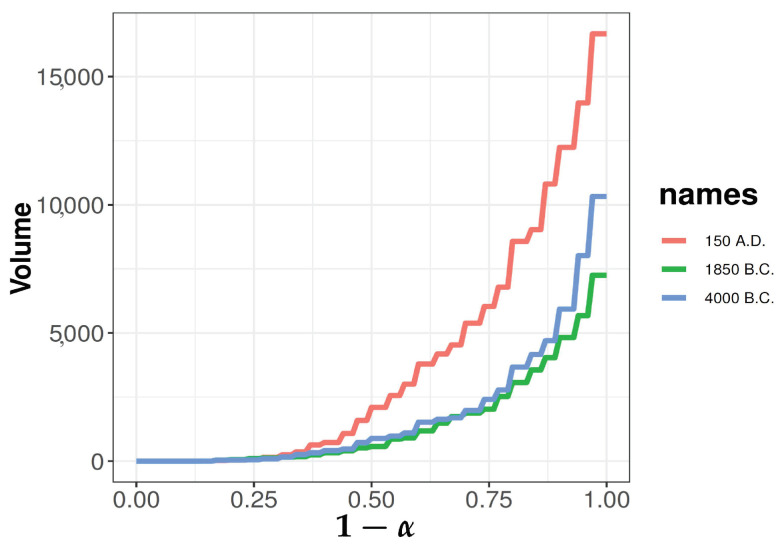
Scale curves of male Egyptian skulls from the last three different time periods under Mahalanobis depth.

The researchers have suggested that changes in skull size over time are evidence of interbreeding between a resident population and a migrant population. The *R* package *HSAUR* includes the male Egyptian skulls data with four measurements (maximal breadth of the skull, basibregmatic height of the skull, basialveolar length of the skull, and nasal height of the skull) from five different time periods: 4000 B.C., 3300 B.C., 1850 B.C., 200 B.C., and 150 A.D. There are 30 measurements for each time period. We wish to determine if there are any differences in the skull sizes in the last three time periods. Figure 9 presents the scale curves of skulls from the last three different time periods under Mahalanobis depth. Since the values of $V(\alpha ;F_{m})$ depend on the units of observations, it is reasonable to have distinct ranges of $V(\alpha ;F_{m})$ in iris and skull datasets.

We can see that the scale curves of skulls from 1850 B.C. and 4000 B.C. are very close. Furthermore, we have estimated the *p*-values for maximum statistic $M_{30,30,30}$ and DbR statistic for each depth function under 10,000 repetitions. Table 1 shows all estimated *p*-values which are greater than 0.05. This confirms that there is no strong evidence to show the interbreeding between a resident population and a migrant population in those three time periods. We note that the asymptotic *p*-value by (13) is 0.027, 0.015, and 0.052 for Mahalanobis, Spatial, and Projection depth, respectively, which are much smaller than the corresponding estimated *p*-values.

## 6. Conclusions

In this paper, we propose two new test statistics for testing the homogeneity of two multivariate samples. Unlike other existing depth-based tests, our proposed test statistics were inspired by the quality index in the context of quality assurance. Constructed based on the pairwise quality indexes, our test statistics are shown to have the same $\chi^{2}\left(1\right)$ asymptotic null distribution. Simulations studies demonstrate the superior performance of the proposed tests. The generalization of the proposed tests into the multivariate multisample situation is discussed as well, along with some simulation studies for three sample comparison. When the number of samples grows, the number of pairwise quality indexes increases. It would also be of interest, although challenging, to consider the higher-order approximation of the asymptotic distributions, as the performance of the proposed tests can be improved.

## Figures and Tables

**Table 1:** Estimated *p*-values for maximum statistic $M_{30,30,30}$ and DbR statistic [14] for each depth function under 10,000 repetitions.

	Maximum Statistic $M_{30,30,30}$	DbR Statistic
Mahalanobis	0.3442	0.1036
Spatial	0.2629	0.0677
Projection	0.3977	0.1457

## Data Availability

Not applicable.

## Appendix A

**Proof** **of Theorem 1.** Define I(x,y,H)=I{D(x;H)⩽D(y;H)}. Under Conditions A1–A4, ref. [6] showed that

$$\begin{array}{cc} \;Q\left(F_{m},G_{n}\right)-Q\left(F,G\right)= & \left(Q\left(F_{m},G_{n}\right)-Q\left(F,G_{n}\right)\right)+\left(Q\left(F,G_{n}\right)-Q\left(F,G\right)\right)\\ \;= & \;\int \int \;I\left(x,y,F\right)dG\left(y\right)d(F_{m}\left(x\right)-F\left(x\right))\\ \;+ & \;\int \int \;I\left(x,y,F\right)dF\left(x\right)d\left(G_{n}\left(y\right)-G\left(y\right)\right)+o_{p}\left(m^{-1/2}\right). \end{array}$$

Let $\xi_{m,n}=\;\int \int \;I\left(x,y,F\right)dG\left(y\right)d\left(F_{m}\left(x\right)-F\left(x\right)\right)+\;\int \int \;I\left(x,y,F\right)dF\left(x\right)d\left(G_{n}\left(y\right)-G\left(y\right)\right)$, and we write $Q\left(F_{m},G_{n}\right)-Q\left(F,G\right)=\xi_{m,n}+o_{p}\left(m^{-1/2}\right)$. Note that *F* and *G* have the same distribution under the null hypothesis, then we have $Q\left(F,G\right)=Q\left(G,F\right)=\frac{1}{2}$ and I(y,x,G)=1−I(x,y,F) by definition of I(x,y,H). Therefore, the pairwise quality indexes possess the following asymptotic symmetry property

(A1) $$Q\left(G_{n},F_{m}\right)-1/2=-\xi_{m,n}+o_{p}\left(n^{-1/2}\right)=1/2-Q\left(F_{m},G_{n}\right)+o_{p}\left(n^{-1/2}\right)+o_{p}\left(m^{-1/2}\right).$$

By [6,7], ${\left[\left(\frac{1}{n}+\frac{1}{m}\right)\frac{1}{12}\right]}^{-1/2}\left\{Q\left(F_{m},G_{n}\right)-1/2\right\}\overset{d}{\to }N\left(0,1\right)$, which means

$${\left[\left(\frac{1}{n}+\frac{1}{m}\right)\frac{1}{12}\right]}^{-1}{\left\{Q\left(F_{m},G_{n}\right)-1/2\right\}}^{2}\overset{d}{\to }\chi^{2}\left(1\right).$$

Since m/n tends to a positive constant, ${\left[\left(\frac{1}{n}+\frac{1}{m}\right)\frac{1}{12}\right]}^{-1/2}\left\{o_{p}\left(n^{-1/2}\right)+o_{p}\left(m^{-1/2}\right)\right\}=o_{p}\left(1\right)$ and ${\left[\left(\frac{1}{n}+\frac{1}{m}\right)\frac{1}{12}\right]}^{-1}\left\{o_{p}\left(n^{-1}\right)+o_{p}\left(m^{-1}\right)+o_{p}\left({\left(mn\right)}^{-1/2}\right)\right\}=o_{p}\left(1\right)$. Further, by (A1),

$${\left[\left(\frac{1}{n}+\frac{1}{m}\right)\frac{1}{12}\right]}^{-1}{\left\{Q\left(G_{n},F_{m}\right)-1/2\right\}}^{2}={\left[\left(\frac{1}{n}+\frac{1}{m}\right)\frac{1}{12}\right]}^{-1}{\left\{Q\left(F_{m},G_{n}\right)-1/2\right\}}^{2}+o_{p}\left(1\right).$$

We can re-express (9) as

$$W_{m,n}\left(w_{1},w_{2}\right)={\left[\frac{1}{12}\left(\frac{1}{m}+\frac{1}{n}\right)\right]}^{-1}{\left\{Q\left(F_{m},G_{n}\right)-\frac{1}{2}\right\}}^{2}+o_{p}\left(1\right),$$

which implies that $W_{m,n}\left(w_{1},w_{2}\right)\overset{d}{\to }\chi^{2}\left(1\right)$. Similarly, we can re-express (10) as

$$M_{m,n}={\left[\frac{1}{12}\left(\frac{1}{m}+\frac{1}{n}\right)\right]}^{-1}{\left\{Q\left(F_{m},G_{n}\right)-\frac{1}{2}\right\}}^{2}+\max\left\{0,o_{p}\left(1\right)\right\},$$

which implies that $M_{m,n}\overset{d}{\to }\chi^{2}\left(1\right)$. □
